# Synthesis of Cobalt-Based Nanoparticles as Catalysts for Methanol Synthesis from CO_2_ Hydrogenation

**DOI:** 10.3390/ma17030697

**Published:** 2024-02-01

**Authors:** Anna Carrasco-García, Seyed Alireza Vali, Zahra Ben-Abbou, Javier Moral-Vico, Ahmad Abo Markeb, Antoni Sánchez

**Affiliations:** 1 Departament of Chemical, Biological and Environmental Engineering, Escola d’Enginyeria, Universitat Autònoma de Barcelona, 08193 Cerdanyola del Vallès, Spain; 2 Departament of Chemistry, Faculty of Science, Assiut University, Assiut 71516, Egypt

**Keywords:** carbon dioxide hydrogenation, methanol synthesis, nanomaterials, heterogeneous catalysis, metal–support interaction

## Abstract

The increasing emission of carbon dioxide into the atmosphere has urged the scientific community to investigate alternatives to alleviate such emissions, being that they are the principal contributor to the greenhouse gas effect. One major alternative is carbon capture and utilization (CCU) toward the production of value-added chemicals using diverse technologies. This work aims at the study of the catalytic potential of different cobalt-derived nanoparticles for methanol synthesis from carbon dioxide hydrogenation. Thanks to its abundance and cost efficacy, cobalt can serve as an economical catalyst compared to noble metal-based catalysts. In this work, we present a systematic comparison among different cobalt and cobalt oxide nanocomposites in terms of their efficiency as catalysts for carbon dioxide hydrogenation to methanol as well as how different supports, zeolites, MnO_2_, and CeO_2_, can enhance their catalytic capacity. The oxygen vacancies in the cerium oxide act as carbon dioxide adsorption and activation sites, which facilitates a higher methanol production yield.

## 1. Introduction

Nowadays, one of the main worldwide concerns is the slow but unstoppable rise in global average temperature, a direct cause of climate change that seems almost unavoidable. The high quantities of greenhouse gases emitted into the atmosphere, of which carbon dioxide emissions are the most important, predict an increase in the Earth’s temperature by 2040 of approximately 1.5 °C compared to the data recorded at the end of the 19th century [1]. Such predictions have alerted the scientific community to develop protocols to lower carbon dioxide emissions, which can be classified according to whether they are carbon capture and storage (CCS) or carbon capture and utilization (CCU) methods. On one hand, CCS methods consist of capturing and storing the gas, and it is so efficient that it could account for almost 20% of the carbon dioxide reduction. Nevertheless, it can be quite costly because industrial-scale installations have to be built. Unfortunately, fossil fuels are still needed as an energy source for this treatment, so carbon dioxide reduction will never be fully completed [2].

On the other hand, CCU results are remarkably interesting since CCU does not only deal with the storage of carbon dioxide but it takes advantage of this gas as a valuable carbon resource for chemical conversion into other products. This is a more viable strategy, as it could not only keep the atmospheric concentration of CO_2_ at acceptable levels but also provide high added-value chemical/fuel products, such as methane and methanol [3,4]. Among all the technologies, carbon dioxide hydrogenation has been considered a promising alternative for obtaining some products from carbon dioxide. An attractive possibility is the production of methanol, a chemical compound that has a wide range of applications. For instance, it can be used as a solvent to obtain several chemicals (formaldehyde, acetic acid, etc.), as well as in the conversion process to olefins, which can be used to produce hydrocarbon fuels and their derivatives that are currently obtained from petroleum [5,6]. 

Carbon dioxide hydrogenation to methanol occurs through two competing reactions. The first step is the methanol synthesis from carbon dioxide and hydrogen:

(1) $$CO_{2}+3H_{2}\to CH_{3}OH+H_{2}O\;\;\;{∆H^{0}}_{298K}=-49.5\;kJ/mol$$

The second step is known as the reverse water–gas shift reaction (RWGS), leading to carbon monoxide production:

(2) $${CO}_{2}+H_{2}\to {CO+H}_{2}O\;\;\;{∆H^{0}}_{298K}=41.2\;kJ/mol$$

In addition, methanol can be indirectly produced from carbon monoxide hydrogenation via the RWGS reaction [7]:

(3) $$CO+2H_{2}\to CH_{3}OH\;\;\;{∆H^{0}}_{298K}=-90.6\;kJ/mol$$

The synthesis of methanol is thermodynamically favored at low temperatures and high pressures [8,9]. 

In recent years, bimetallic catalysts have been elaborately studied owing to their chemical, electronic, and structural characteristics. Additionally, the synergy between metals leads to the generation of a catalytic system, which, unlike monometallic systems, presents advantages in terms of catalytic activity, methanol selectivity, and catalyst stability [10]. The catalysts for carbon dioxide hydrogenation to methanol studied more comprehensively include Cu-Zn-based catalysts, although other bimetallic catalysts such as Pd-Zn, Pd-Ga, Cu-Ni, and Ni-Ga have been investigated as well [7]. In addition to these mentioned catalysts, methanol has also been obtained using cobalt-based catalysts. Stangeland et al. [11] demonstrated that Co_3_O_4_/MnOx was efficient in the production of methanol at mild pressures, but a variety of by-products were also obtained. These authors achieved a manifold increase in methanol yield with Co_3_O_4_/MnOx catalysts compared to Cu/Zn-based catalysts under similar reaction conditions [11]. Wang et al. [12] investigated silica-supported cobalt catalysts with the aim of accelerating the selectivity of methanol obtained from the carbon dioxide hydrogenation, and they showed that silica incorporation in the cobalt catalysts improved both carbon dioxide conversion and selectivity toward methanol. 

One of the significant features of cobalt-based catalysts is their potential to catalyze various carbon dioxide conversion reactions such as methanation [13,14], synthesis of higher alcohols [15,16], or methanol synthesis [11,12,17,18]. In these investigations, it was shown that the selectivity of these catalysts can be impacted by the use of different supports. In the present study, different supports including zeolite, manganese oxide, and cerium oxide have been investigated to increase the catalytic activity of cobalt-based catalysts. It is hypothesized that different metal–support interactions between cobalt nanoparticles and the support will enhance the catalytic sites, leading to higher methanol yield and selectivity. Zeolites with a microporous three-dimensional structure based on SiO_4_ and AlO_4_ present substantial catalytic and adsorption properties [19]. Cerium oxide is a non-toxic oxide, which is of great importance in catalysis due to its ability to store and supply oxygen [20,21]. It provides a large number of oxygen vacancies on the surface, which can function as sites for the adsorption and activation of carbon dioxide. Manganese oxide is a mesoporous support and its effect as support for nanocatalysts used for carbon dioxide hydrogenation to methanol has not been reported [11]. 

Capping agents are reported to be significant stabilizers since they counteract the attraction between nanoparticles, thus inhibiting their overgrowth and aggregation. These agents are amphipathic molecules that are characterized by having a polar head group and a non-polar tail, and due to this amphipathic property, they improve compatibility with other phases. Different types of protection agents have been implemented in the synthesis of nanomaterials (surfactants, polymers, polysaccharides, etc.), aiming to obtain nanoparticles of smaller sizes, which will lead to the highest surface area of nanoparticles [22,23]. In this study, the agglomeration of the synthesized nanoparticles was controlled by steric stabilization using polyvinylpyrrolidone (PVP). 

This work is centered on studying the catalytic activity of supported and unsupported cobalt-based nanoparticles. The aim is to evaluate this type of nanomaterial in order to obtain high methanol production yields and high selectivity under mild pressure and temperature conditions. 

## 2. Materials and Methods

### 2.1. Materials

Sodium borohydride (NaBH_4_) (99.0%), cobalt (II) chloride hexahydrate (CoCl_2_·6H_2_O) (99.0%), polyvinylpyrrolidone (PVP), sodium carbonate (Na_2_CO_3_) (99.5%), zeolite, cerium (III) nitrate hexahydrate (Ce(NO_3_)_3_·6H_2_O (99.0%), sodium hydroxide (NaOH) (98.0%), manganese (II) sulfate monohydrate (MnSO_4_·H_2_O) (99.0%), and potassium permanganate (KMnO_4_) (99.5%) were all purchased from Sigma-Aldrich (Barcelona, Spain). A mixed-gas bottle of carbon dioxide and hydrogen with a molar ratio of 1:3, respectively, was provided by Carburos Metálicos S.A. (Barcelona, Spain).

### 2.2. Synthesis of Nanocomposites

#### 2.2.1. Co nanoparticles Synthesis

Co nanoparticles were synthesized by the chemical reduction method. A total of 1.10 g of cobalt (II) chloride hexahydrate was dissolved in 100 mL of deionized water with magnetic stirring and nitrogen bubbling to avoid the oxidation of cobalt. Then, a solution of sodium borohydride (0.25 M) was added dropwise, and the solution was left to stir for 20 min to ensure all the cobalt was reduced [24]. Once the reaction was complete, the pH reached 9.3, and cobalt nanoparticles were separated using a magnet. The product was washed three times with deionized water to remove any impurities and dried in an oven at 105 °C overnight. Co nanoparticles with PVP were synthesized via a similar route by adding 1.10 g of PVP to the cobalt (II) chloride hexahydrate solution. 

#### 2.2.2. Co_3_O_4_ Nanoparticles Synthesis

Co_3_O_4_ nanoparticles were synthesized via the co-precipitation method. Briefly, 0.61 g of cobalt (II) chloride hexahydrate was dissolved in 100 mL of deionized water using magnetic stirring for 20 min. The synthesis was performed in a 500 mL Scharlau Minireactor HME-R/500 with mechanical stirring and heating at constant ambient pressure. Cobalt (II) chloride hexahydrate solution was added to the reactor and the precipitation agent, sodium carbonate solution (1 M), was added dropwise at a flow rate of 5 mL/min using a peristaltic pump (Watson Marlow SCI 400, Watson-Marlow GmbH, Rommerskirchen, Germany), and the solution was left to age at 60 °C for 5 h with constant stirring at 120 rpm. The Co_3_O_4_ nanoparticles were obtained after centrifuging three times for 15 min at 5000 rpm and drying at 105 °C overnight [24]. Co_3_O_4_ nanoparticles with PVP were synthesized via a similar route by adding 0.62 g PVP to the cobalt (II) chloride hexahydrate solution. 

#### 2.2.3. MnO_2_ Nanoparticles Synthesis

The co-precipitation method was also employed for the synthesis of MnO_2_ nanoparticles. A total of 3.12 g of manganese (II) sulfate monohydrate was dissolved in 100 mL deionized water. The potassium permanganate solution (0.15 M) was added to the above solution dropwise, and the mixture was vigorously stirred in a Scharlau Minireactor HME-R/500 (Barcelona, Spain) at 80 °C for 5 h. Then, a sodium hydroxide solution was added dropwise to adjust the pH to 11. Afterwards, the nanoparticles were centrifuged and washed three times with deionized water. Finally, the nanoparticles obtained were dried at 105 °C for 12 h [25].

#### 2.2.4. Co_3_O_4_/Zeolite Nanocomposite Synthesis

To immobilize the Co_3_O_4_ nanoparticles onto zeolite, the same Co_3_O_4_ synthesis described above was followed. In 200 mL of ultrapure water, 1 g of zeolite was dispersed in an ultrasound bath for 15 min. Once zeolite was dispersed, it was transferred to the reactor, and the same Co_3_O_4_ synthesis procedure was performed.

#### 2.2.5. CeO_2_ Nanoparticle Synthesis

CeO_2_ nanoparticles were synthesized through a co-precipitation method as well. A total of 5.12 g of cerium (III) nitrate hexahydrate was dissolved in another 100 mL of deionized water. Then, 1.88 g of the precipitant agent, sodium hydroxide, was dissolved in 100 mL of deionized water and was added dropwise to cerium solution in a reactor (Scharlau Minireactor HME-R/500, Scharlab, Barcelona, Spain) with mechanical agitation using a peristaltic pump at 7 mL/min. The solution was then left for 15 min under stirring. Eventually, the precipitates obtained were centrifuged, washed with deionized water three times, and then dried at 105 °C overnight [26].

#### 2.2.6. Co_3_O_4_/CeO_2_ Nanocomposite Synthesis

To immobilize the Co_3_O_4_ nanoparticles onto CeO_2_, the same Co_3_O_4_ synthesis procedure was followed. In 200 mL of deionized water, the nanoparticles of CeO_2_ were scattered in an ultrasound bath for 15 min. Once dispersed, the same Co_3_O_4_ synthesis procedure was carried out, but first, the solution of the CeO_2_ nanoparticles was transferred to the reactor. The resulting weight ratio of Co_3_O_4_ nanoparticles and CeO_2_ supports was 2:1 (g/g), respectively. 

#### 2.2.7. Co_3_O_4_/MnO_2_ Nanocomposite Synthesis

Co_3_O_4_ nanocomposites were synthesized using the co-precipitation method. The same Co_3_O_4_ synthesis procedure followed, but first, the previously synthesized MnO_2_ nanoparticles were dispersed in 200 mL of deionized water in an ultrasound bath for 15 min. Subsequently, the same Co_3_O_4_ synthesis was followed after MnO_2_ was added to the reactor. The resulting weight ratio of Co_3_O_4_ nanoparticles and MnO_2_ supports was 2:1 (g/g), respectively.

### 2.3. Characterization of Catalysts

X-ray diffraction (XRD) was used to perform a structural analysis of the nanoparticles and their crystallographic structure. All the analyses were conducted after the materials had been thermally treated. A diffractometer (PANalytical X’Pert, Malvern Panalytical, Malvern, UK) using Cu-Kα radiation was employed to record the X-ray diffraction patterns. The measurements were conducted at room temperature in a range of 10.0–80.0° on 2θ with a step size of 0.026°. The data analysis was completed by simulating the nanoparticles’ crystallinity with the X’Pert High Score (PANalytical) software (Version 3.0.5). A scanning electron microscope (SEM) (FEI Quanta 650F ESEM, FEI, Hillsboro, OR, USA) equipped with an energy-dispersive spectroscopy (EDS) source was used to determine the morphology, size distribution, and composition of the nanoparticles. Samples were prepared on copper and graphite grids (TED PELLA, Inc., Redding, CA, USA). The microstructure, the size, morphology, and size distribution of the nanoparticles were determined using a transmission electron microscope (TEM) (FEI TECNAI G2 F20, FEI, Hillsboro, OR, USA). The samples were analyzed using copper grids (TED PELLA, Inc., Redding, CA, USA). An AutoChem (Micromeritics) instrument using 12 vol% H_2_/Ar at a flow of 50 N mL·min^−1^ in a temperature range of 35−800 °C at a heating ramp of 10 °C·min^−1^ was used for temperature-programmed reduction (H_2_-TPR) measurements. The amount of H_2_ uptake was measured with a thermal conductivity detector. A total of 100 mg of the sample was used for each measurement.

### 2.4. Catalytic Activity Test

The catalytic test was carried out in a stainless-steel fixed-bed reactor. Prior to the tests, samples were calcinated at 500 °C for 4 h, and those containing elemental cobalt were reduced by a hydrogen flow of 40 mL/min at 350 °C for 2 h. The catalyst samples were fixed between two layers of glass wool at each end of the reactor. The catalytic tests were carried out under two moderate pressure values of 10 and 15 bar. The flow rate of the stoichiometric H_2_/CO_2_ mixture was 10 mL/min. To study the impact of reaction temperature on the catalytic activity, the reaction was performed at temperatures ranging from 180 to 280 °C. After fixing each temperature, catalysts were stabilized for half an hour, resulting in a total time of operation of more than four hours. Sampling bags (SKC FlexFoil PLUS Sample Bag, SKC, Seoul, Republic of Korea) were utilized to collect the gas samples, and methanol was measured in a gas chromatograph (Shimadzu GC-2010, Shimadzu, Kyoto, Japan) with a flame ionization detector (FID) using helium as carrier gas. The software used was Chromeleon (Version 6.80 SR5b), the inlet temperature was 260 °C, and the flow was 50 mL/min; the detector temperature was 280 °C. An Agilent 7890B chromatograph (Agilent, Santa Clara, CA, USA) was used to measure carbon monoxide and dioxide, employing a thermal conductivity detector (TCD) and helium as the carrier gas. The software used was OpenLab (Version A.01.04), the inlet temperature was 120 °C, the inlet flow was 20 mL/min, and the detector temperature was 150 °C. To study the catalytic activity, methanol space–time yield (STY), as well as methanol selectivity, were calculated according to the following equations. Carbon monoxide was observed to be the only side-product of the reaction.

(4) $${CH}_{3}OH\;STY\left(\frac{g}{kg_{cat}\times h}\right)=\left(\frac{Mass\;of\;methanol\;\left(g\right)\;formed}{W_{cat}\left(kg\right)\times Hour}\right)$$

(5) $${CH}_{3}OH\;Selectivity\left(\%\right)=\left(\frac{moles\;of\;methanol\;formed}{n{\left[{CO}_{2}\right]}_{in}-n{\left[{CO}_{2}\right]}_{out}}\right)\times 100$$

## 3. Results and Discussion 

### 3.1. Structural and Morphological Characterization of Nanomaterials

**Figure 1:**
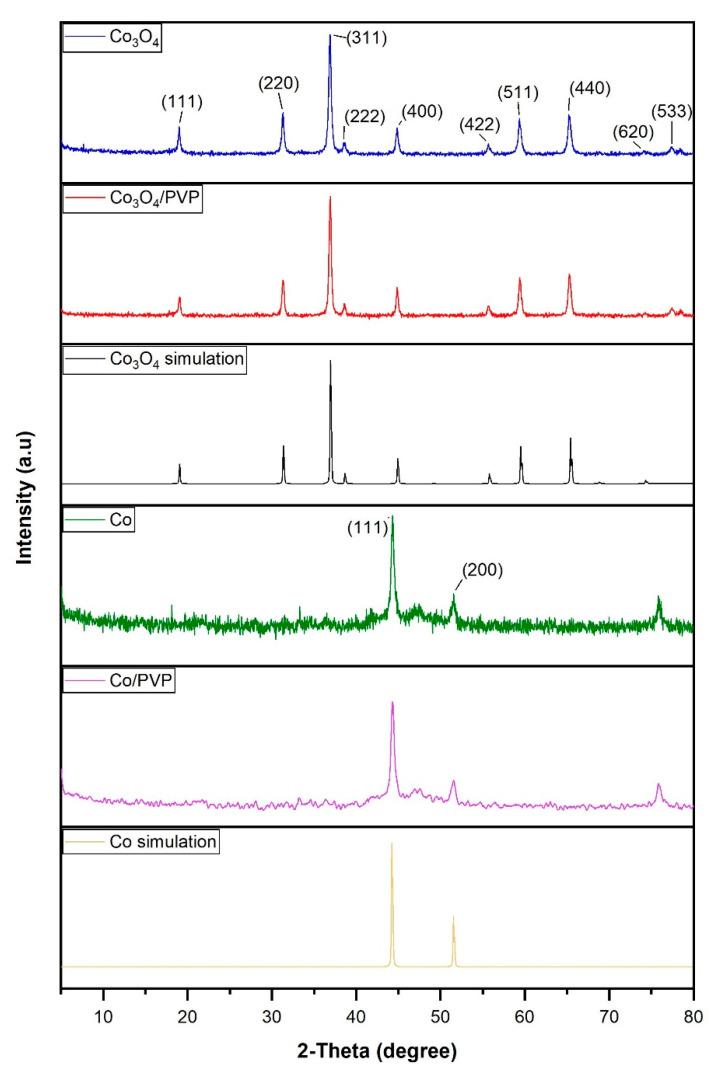
XRD patterns of the following samples and simulations: Co simulation; Co nanoparticles; Co/PVP nanoparticles; Co_3_O_4_ nanoparticles; Co_3_O_4_/PVP nanoparticles; Co_3_O_4_ simulation.

The XRD patterns obtained for cobalt and cobalt oxide with and without capping agents (PVP) and their corresponding simulations using PANALYTICAL X’Pert High Score software are presented in Figure 1. As is shown, the peaks at diffraction angles of 2θ of 19.05°, 31.27°, 36.90°, 38.56°, 44.82°, 55.70°, 59.40°, 65.30°, 74.14°, and 77.14° correspond to the (111), (220), (311), (222), (400), (422), (511), (440), (620), and (533) planes of Co_3_O_4_ [11,23,27]. Furthermore, the peaks at 2θ of 44.23° (111) and 51.52° (200) corresponded to Co nanoparticles [24]. It can also be observed that the XRD spectra of the synthesized materials are very similar to the simulation, which means a high level of purity and crystallinity. Regarding the Co_3_O_4_ and Co with and without PVP, it was observed that PVP did not interfere with the crystallinity of cobalt and cobalt oxide nanoparticles. As can be seen, there is no presence of cobalt oxide in the cobalt XRD patterns, indicating that the synthesis of cobalt nanoparticles was satisfactory, and all the nanoparticles were completely reduced. 

**Figure 2:**
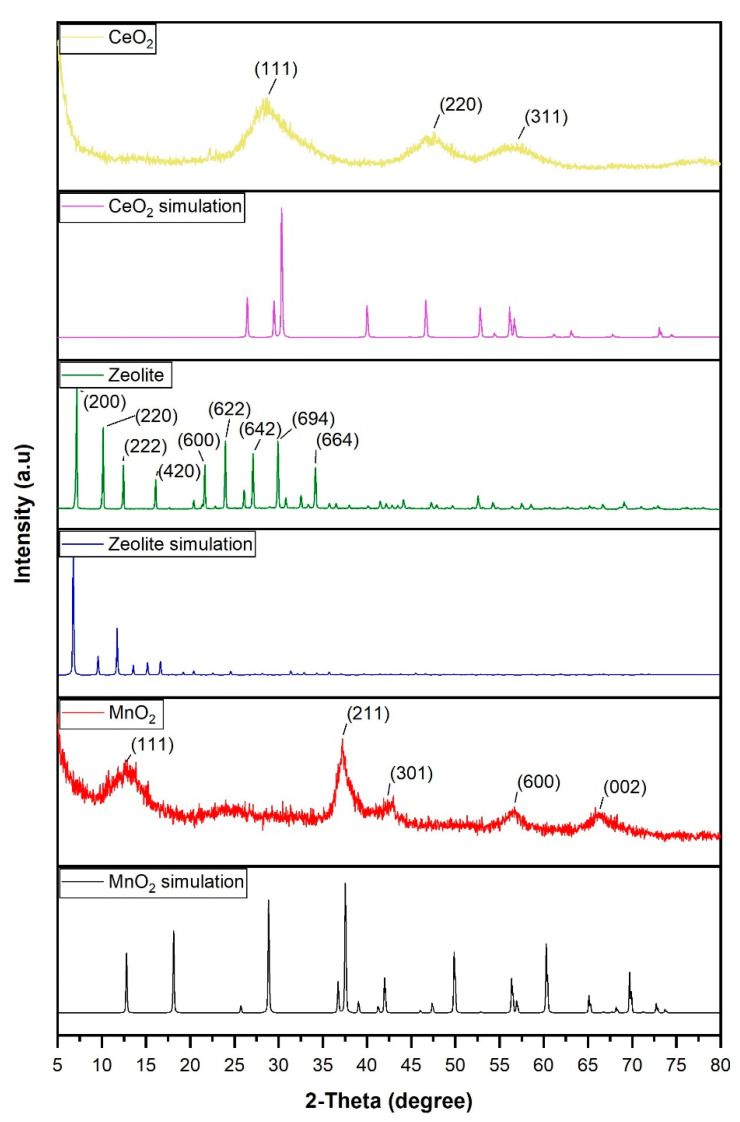
XRD patterns of the following samples and simulations: CeO_2_; CeO_2_ simulation; zeolite; zeolite simulation; MnO_2_; MnO_2_ simulation.

The X-ray diffraction patterns of all the supports are shown in Figure 2. Regarding two of the supports, CeO_2_ and MnO_2_, it is observed that not all the peaks observed in the simulation correspond to the peaks observed in the synthesized sample, which indicates that these samples are not completely crystalline. However, the peaks obtained for the zeolite sample show a more crystalline structure, with a spectrum that is more similar to the simulation than in the case of the other two supports. 

**Figure 3:**
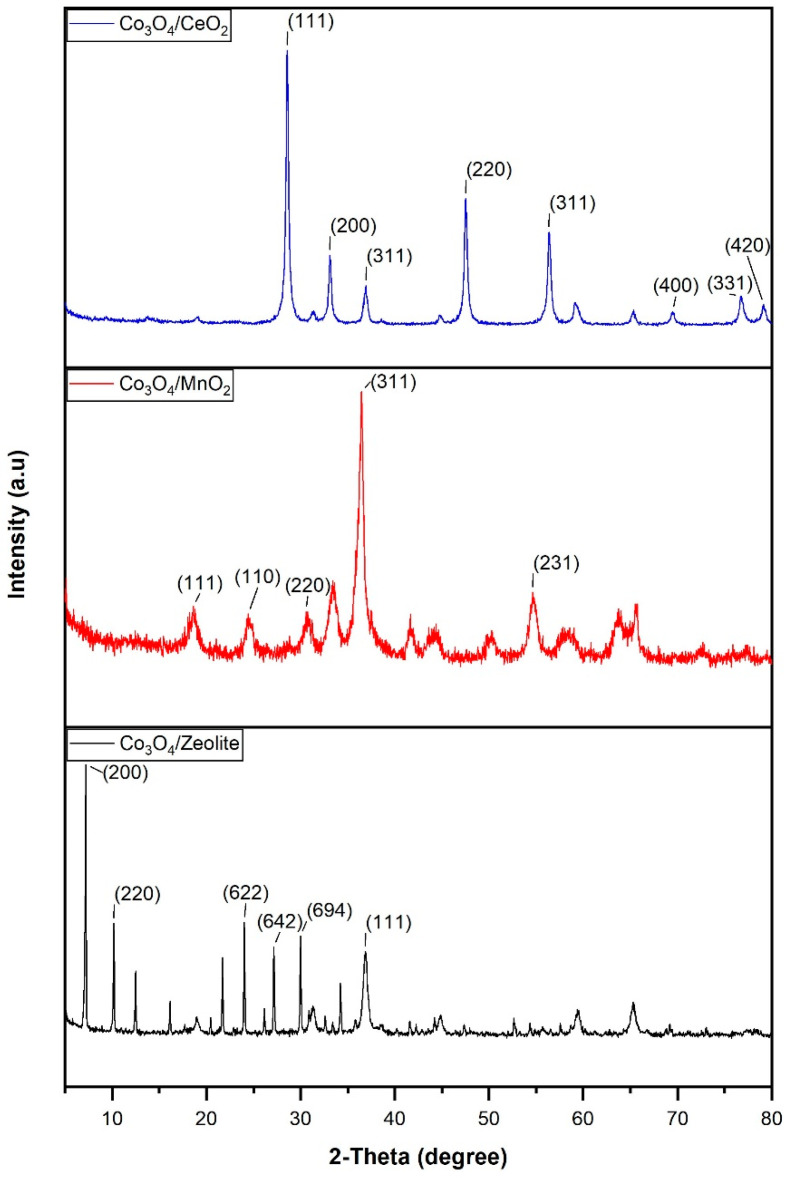
XRD patterns of the following samples: Co_3_O_4_/CeO_2_; Co_3_O_4_/MnO_2_; Co_3_O_4_/zeolite.

The X-ray diffraction patterns of the nanoparticles and their supports are shown in Figure 3. Regarding the highest peaks of Co_3_O_4_/zeolite, it is observed that the first peaks are associated with zeolite since the X’Pert High Score software determined that they correspond to the two typical elements of zeolite (aluminum and silicon) [28]. Small peaks corresponding to Co_3_O_4_ were also observed at the following angles: 19.05°, 31.27°, 36.90°, 38.56°, 44.82°, 55.70°, 59.40°, and 65.30°. Regarding the XRD patterns of Co_3_O_4_ /CeO_2_, it is observed that the peaks situated at the angles of 28.50°, 33.10°, 47.50°, 56.30°, 69.40°, 76.70°, and 79.10° belong to CeO_2_. They are associated with planes (111), (200), (220), (311), (400), (331), and (420), respectively (Figure 3) [27]. Moreover, other characteristic peaks of Co_3_O_4_ are seen at angles 19.05°, 31.27°, 36.90°, 38.56°, 44.82°, 59.4°, and 65.30° (Figure 3) [29,30]. Finally, in the Co_3_O_4_/MnO_2_ patterns, the peaks of both materials can also be visualized (Figure 3). The angles 24.50°, 41.60°, 50.30°, 54.70°, 63.70°, 72.30°, and 79.19° correspond to Co_3_O_4_/MnO_2_ and are related to the (110), (120), (220), (231), (130), (343), and (330) planes (Figure 3) [31,32]. Another wide peak around 58° is probably the peak at 59.4° attributed to Co_3_O_4_, which is broadened due to the interaction with MnO_2_. The other profiles observed correspond to the cobalt oxides present in the sample since they coincide with the Co_3_O_4_ angles described above. This means that there is a coexistence of elemental cobalt and its oxidized species in the sample.

**Figure 4:**
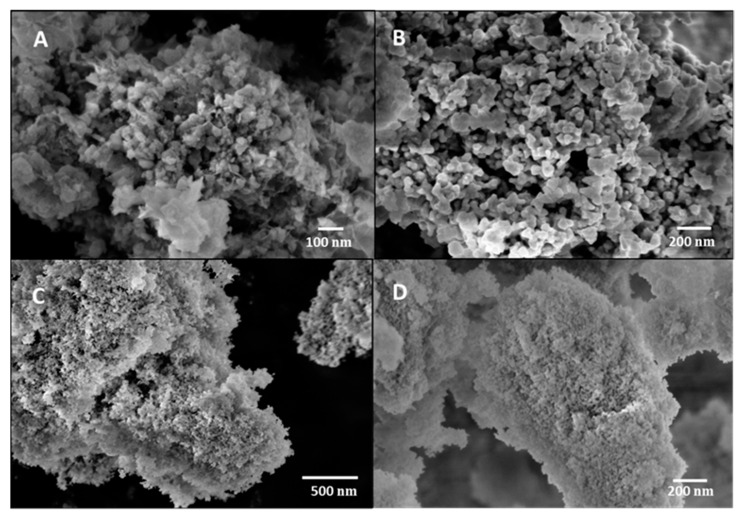
SEM images obtained from the catalyst samples: (**A**) Co nanoparticles; (**B**) Co/PVP nanoparticles; (**C**) Co_3_O_4_ nanoparticles; (**D**) Co_3_O_4_/PVP nanoparticles.

**Figure 5:**
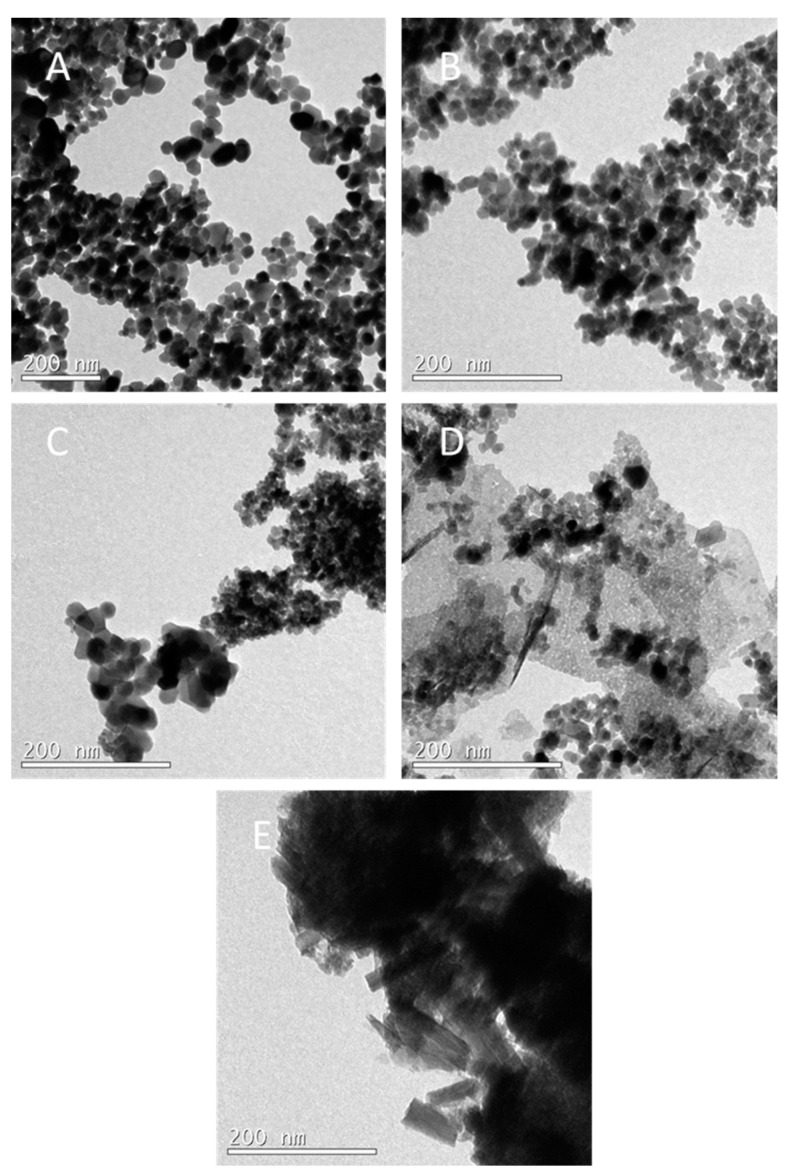
TEM images obtained from the catalyst samples: (**A**) Co_3_O_4_; (**B**) Co_3_O_4_/PVP; (**C**) Co_3_O_4_/CeO_2_; (**D**) Co_3_O_4_/zeolite; (**E**) Co_3_O_4_/MnO_2_.

The characterization and morphology study of the materials was performed using a scanning electron microscope (SEM) and a transmission electron microscope (TEM). Figure 4C shows the SEM image of the Co_3_O_4_ nanoparticles, which presents an irregular shape with a high degree of agglomeration, as previously reported [24]. Nevertheless, when PVP was added, the nanoparticles were smaller and with a more spherical geometry (Figure 4D). The nanoparticle sizes were determined by analyzing TEM images with ImageJ software (Version 1.46r) (Figure 5). The mean size for Co_3_O_4_ nanoparticles was 27 ± 5 nm (Figure 5B), whereas when PVP was used, the size was reduced to 15 ± 2.5 nm (Figure 5B,E), confirming the observation of SEM images. It is reported in the literature that by adding a capping agent, the nanoparticles are smaller, and this effect leads to an increase in the surface area of the catalyst, which means that the active sites are more exposed [22,23]. Therefore, the catalytic activity of the catalyst increases. The morphology of cobalt nanoparticles with and without PVP is shown in Figure 4A,B, respectively. The same effect is observed when PVP was added to the synthesized material: the morphology obtained is well defined and the particles are smaller. 

**Figure 6:**
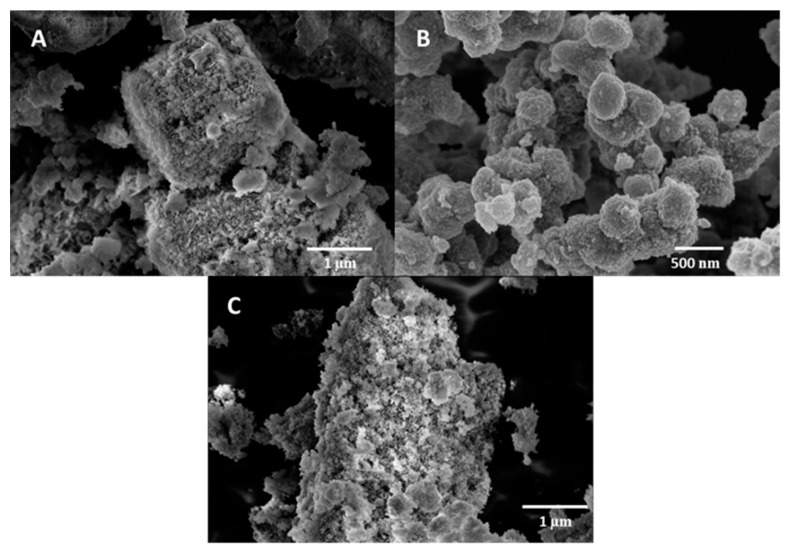
SEM images obtained from the catalyst samples: (**A**) Co_3_O_4_/zeolite; (**B**) Co_3_O_4_/MnO_2_; (**C**) Co_3_O_4_/CeO_2_.

SEM images of nanoparticles embedded in a support are presented in Figure 6. Co_3_O_4_ nanoparticles immobilized on zeolite show a granular morphology (Figure 6A). The TEM image of the same sample shows a good dispersion of cobalt oxide nanoparticles, and a mean size of 13 ± 1.7 nm (Figure 5D), which means that these cobalt oxide nanoparticles are even smaller than those synthesized in the presence of PVP. It is reported in the literature that the use of a support helps to obtain nanoparticles with better distribution and a higher surface area [30]. In Figure 6C, Co_3_O_4_ nanoparticles supported on CeO_2_ can be observed, demonstrating that the nanoparticles have a very small size and a good distribution. Indeed, the Co_3_O_4_ nanoparticles supported on CeO_2_ analyzed with TEM (Figure 5C) reveal a size of 22.6 ± 4.2 nm for the former, which is slightly smaller than unsupported Co_3_O_4_ (27 ± 5 nm). Figure 5C also shows a good interaction between cobalt and cerium oxide nanoparticles. 

Co_3_O_4_ nanoparticles embedded in MnO_2_ show an aggregated morphology, as previously reported in the literature (Figure 6B) [25]. This fact is also shown in the TEM image of the same sample (Figure 5E).

The elemental composition of the catalyst samples was determined by energy dispersive spectrometry (EDX). This analysis was carried out on the nanoparticles immobilized on the three tested different supports in order to confirm the presence of the expected elements and possible impurities. The content of the latter is expressed in the “others” row in Table 1, Table 2 and Table 3 and includes mainly chloride, sodium, and potassium coming from the synthesis process. As observed with XRD, the EDX spectrum of Co_3_O_4_/zeolite confirms the presence of expected chemical elements (Co, Al, Si) (Table 3) [33]. Nevertheless, a very small presence of impurities from the reducing agent used during the synthesis was also detected, indicating that the material should have been washed more times. Co and Ce were detected in the Co_3_O_4_/CeO_2_ sample as expected (Table 2). In the Co_3_O_4_/MnO_2_ sample (Table 1), the presence of Co and Mn elements was detected, as well as a very small amount of sodium and potassium. 

**Figure 7:**
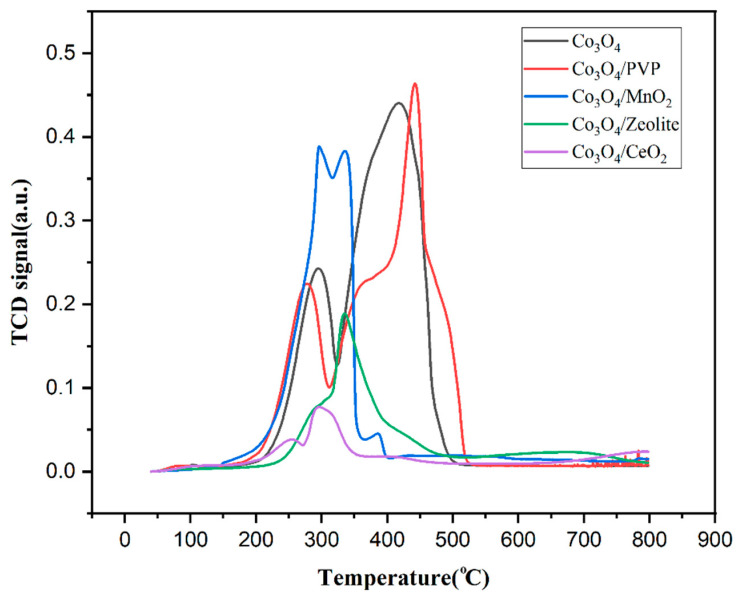
H_2_-TPR profiles of the following samples: Co_3_O_4_; Co_3_O_4_/PVP; Co_3_O_4_/MnO_2_; Co_3_O_4_/zeolite; and Co_3_O_4_/CeO_2_.

The reducibility of the Co_3_O_4_ catalyst supported on different supports was investigated by hydrogen temperature-programmed reduction (H_2_-TPR) in a 50–800 °C temperature range. The corresponding TPR profiles are shown in Figure 7. As can be observed, a different reduction behavior is obtained for the Co_3_O_4_ when supported on different materials as a result of the metal–support interaction between Co_3_O_4_ and the support. The reduction behavior of all samples can be seen to consist of two main peaks. The first peak at lower temperatures (250–300 °C) can be attributed to the well-dispersed Co_3_O_4_, while the peaks at higher temperatures (400–500 °C) correspond to the reduction in bulk Co_3_O_4_. In general, MnO_2_ seems to have had the most constructive effect on the reducibility of Co_3_O_4_ since the peak corresponding to bulk Co_3_O_4_ disappeared and the peaks at lower temperatures corresponding to the well-dispersed Co_3_O_4_ strengthened significantly in the TPR profile of Co_3_O_4_/MnO_2_, demonstrating a strong metal–support interaction between Co_3_O_4_ and MnO_2_, leading to better dispersion of Co_3_O_4_ nanoparticles. However, this result was not supported by TEM images, as previously discussed.

Zeolite can be seen to have also affected the reduction behavior of Co_3_O_4_, as there is a broad peak at 250–500 °C for the sample Co_3_O_4_/zeolite, while no peak at higher temperatures can be observed. In addition, CeO_2_ support seems to have had a slightly positive effect on the reducibility of Co_3_O_4_, as the peak at higher temperatures in the TPR profile of Co_3_O_4_/CeO_2_ also disappeared, while two peaks appeared at 250–350 °C. Finally, comparing the profile of Co_3_O_4_ and Co_3_O_4_/PVP, although there can be seen a slight shift to lower temperatures for the first peak related to dispersed Co_3_O_4_ and a slight shift to higher temperatures for the peak related to bulk Co_3_O_4_, it can be stated that there is no significant change in the reducibility of Co_3_O_4_ when synthesized using PVP, showing that the metal–support interaction can be considered negligible for the catalyst Co_3_O_4_/PVP.

### 3.2. Catalytic Activity of the Catalysts 

**Figure 8:**
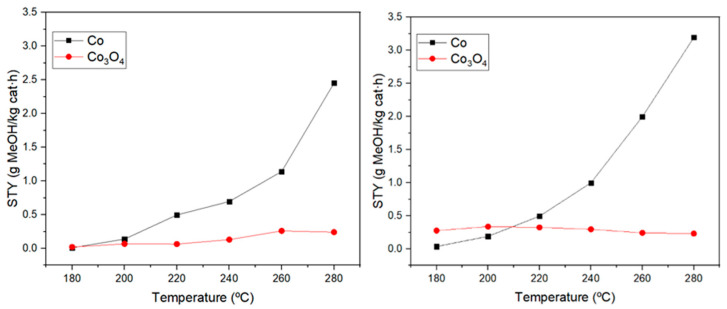
STY values for cobalt and cobalt oxide nanoparticles at a pressure of 10 bar (**left**) and 15 bar (**right**).

To study the catalytic activity of the samples, methanol STY and selectivity were obtained. Figure 8 presents methanol STY for cobalt and its oxide as a function of operating temperature at pressures of 10 and 15 bar, respectively. The error bars have not been included in the STY figures due to their low values. The effect of the reaction temperature was also investigated, and it can be seen that for cobalt samples methanol STY increases gradually as the temperature rises. However, for the Co_3_O_4_ catalyst, this increase was not so evident operating at 10 bar, and when the operating pressure was 15 bar, a slight decrease in the STY values as the temperature rises can be detected. On the contrary, in the case of cobalt, the results showed that a better catalytic activity is obtained at 15 bar, which is an expected result, as the use of high pressures is advantageous due to the exothermic nature of the reaction [8,11]. 

Furthermore, the catalytic activity of cobalt and cobalt oxide was compared in terms of STY and selectivity to understand which cobalt species presents the active sites more favorable for methanol synthesis. As can be observed in Figure 8, cobalt nanoparticles give a methanol STY of 3.2 g·kg_catalyst_^−1^h^−1^, while only 0.25 g·kg_catalyst_^−1^h^−1^ was obtained for cobalt oxide nanoparticles. Hence, metallic cobalt possesses active sites catalyzing the methanol synthesis from the carbon dioxide hydrogenation reaction more efficiently. This is due to the fact that the selectivity of the reaction is much more favored for methanol formation than for carbon monoxide production when cobalt is used instead of Co_3_O_4_ because the latter material is highly selective toward methane and carbon monoxide formation [34,35]. In many studies, this effect has been attributed to a lower carbon dioxide adsorption on the cobalt surface compared to Co_3_O_4_, which favors the formation of products, such as methane, when using the oxide [36]. However, the catalyst selected for this study was cobalt oxide due to its high stability compared to elemental cobalt [37].

**Figure 9:**
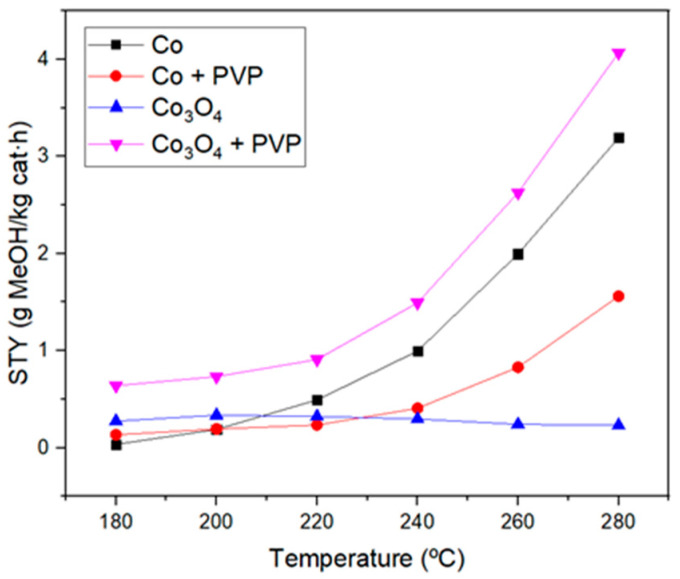
Methanol STY values obtained for cobalt and cobalt oxide nanoparticles with and without PVP at 15 bar.

Two ways to improve the catalytic activity working with cobalt oxide nanoparticles were studied: (a) the addition of polyvinylpyrrolidone (PVP), in order to restrain the overgrowth of the nanoparticles, and (b) the addition of a support, to improve the synergistic effect between the nanoparticles and the support. A comparison of the catalyst’s performance with and without PVP was carried out to analyze the effect of this polymer on the materials. The results are shown in Figure 9. By adding PVP in the synthesis of Co_3_O_4_, higher methanol STY was obtained, which is accounted for by the smaller size of Co_3_O_4_ nanoparticles, showing a more spherical morphology (Figure 4C,D). This aspect leads to a higher specific surface area and, therefore, more availability of the catalytic sites. 

**Figure 10:**
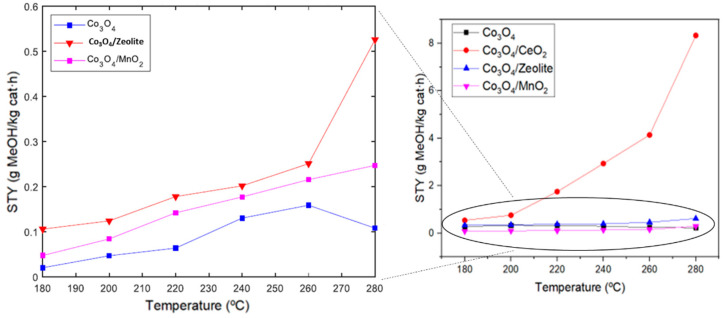
Methanol STY values obtained for samples at different temperatures and a pressure of 15 bar.

In order to investigate the impact of supports on the catalytic activity of Co_3_O_4_ nanoparticles, they were immobilized on different supports including zeolite, CeO_2_, and MnO_2_. As seen in Figure 10, more methanol STY was obtained when immobilizing Co_3_O_4_ on CeO_2_. This can be attributed to the strong metal–support interaction between Co_3_O_4_ and CeO_2_, which generates interfacial sites that can synergistically catalyze the methanol synthesis reaction. In addition, oxygen vacancies present in CeO_2_ are also assumed to facilitate the adsorption and activation of carbon dioxide [8,20,21,38]. The other support studied was zeolite, which resulted in an improvement of methanol production since the presence of aluminum atoms in these silicate-based materials provides negative charges that are compensated by exchangeable cations in the pore space, and these porous characteristics in the zeolite structure are those that allow greater carbon dioxide adsorption capacity [22,37,39]. Co_3_O_4_ immobilized on MnO_2_ also resulted in more methanol STY compared to Co_3_O_4_. This is probably a result of the interactional effect of the two materials, as the catalytic activities of the individual materials are lower and less selective for methanol (Figure 8), indicating the importance of the architecture and nature of the interface [11]. Comparing the methanol STY of the three supports studied, CeO_2_ revealed the best results as a support of Co_3_O_4_, which can be due to the oxygen vacancies promoting carbon dioxide adsorption and activation, as well as the generation of the interfacial sites between CeO_2_ and Co_3_O_4_, hence favoring the methanol synthesis reaction. Cobalt oxides have previously been reported to provide a low methanol production yield when compared with supported or modified cobalt compounds. For instance, Wang et al. [12] show that unsupported cobalt oxide shows the lowest yield compared with these species supported on SiO_2_ or modified through linkages with silicon. Li et al. [40] show a similar result when comparing manganese oxide supported on cobalt oxide. Although the results make it difficult to compare the catalytic performance of the compounds studied in this work with others in the literature due to the different experimental conditions and material features, it has been previously reported that cobalt oxide species, like In_2_O_3_, supported on Co_3_O_4_ have a catalytic activity that reveals an STY of up to 650 g·kg_catalyst_^−1^h^−1^ [41]. Another similar compound, a cobalt–indium composite obtained by pyrolysis, was evaluated by Wang et al. to perform an STY of 620 g·kg_catalyst_^−1^h^−1^ [42]. However, the compounds studied in the mentioned works are more complex structures, such as a cobalt metal–organic framework impregnated with indium, and a pyrolytic composite of cobalt and indium (Co_3_InC_0.75_-In_2_O_3_), respectively. Also, the pressures used are much higher; they are 50 bar. Other bimetallic catalysts using noble metals, like palladium, in particular Pd/Zn materials supported on carbon nanotubes, have also obtained high STY values of 371 g·kg_catalyst_^−1^h^−1^ but at higher pressures of 30 bar [43]. The Co_3_O_4/_CeO_2_ catalyst analyzed in this study improved the catalytic performance of a material composed of copper and zinc, which are the typical elements used for this catalysis reaction, Cu/ZnO/zeolite, reported by Carrasco García et al. [22]. In particular, the latter obtained an STY of 4.3 g·kg_catalyst_^−1^h^−1^ and Co_3_O_4/_CeO_2_ an STY of 8.3 g·kg_catalyst_^−1^h^−1^ at the same temperature and pressure conditions. 

**Figure 11:**
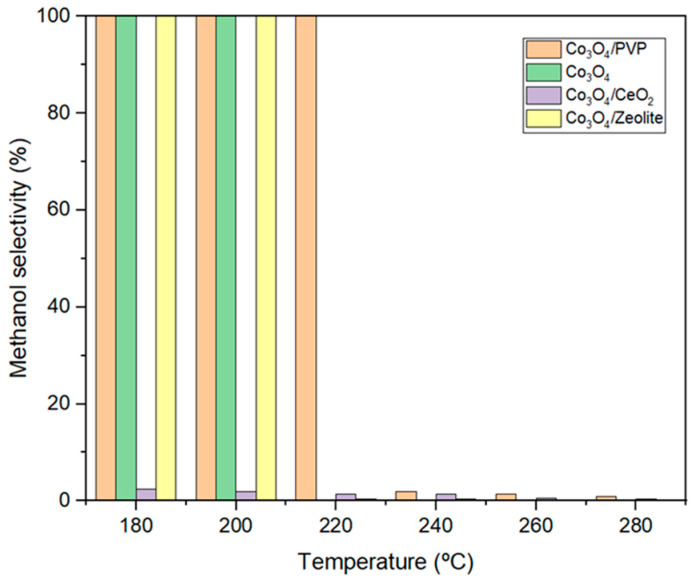
Methanol selectivity (%) values at 15 bar of the best catalysts.

Methanol selectivity for the catalyst samples is presented in Figure 11. As can be seen, at 180 °C and 15 bar, the methanol selectivity for all samples was 100%, except for Co_3_O_4/_CeO_2_, indicating that no carbon monoxide was formed at this temperature using the indicated materials. Nonetheless, with the temperature increase, methanol selectivity decreases because the change in the enthalpy of methanol synthesis is negative, and, therefore, it is an exothermic reaction, which is more favored at lower temperatures (Figure 11) [9,44]. In the case of Co_3_O_4_ with PVP and Co_3_O_4_/zeolite, a decrease in selectivity toward methanol was only observed at 220 °C. For the other remaining catalysts, this methanol selectivity decreased considerably because of the production of carbon monoxide at high temperatures (Figure 11) [11,40].

## 4. Conclusions 

In this work, a series of cobalt-based catalysts with different supports were synthesized, and their catalytic activity for methanol production from carbon dioxide hydrogenation was studied. XRD analysis detected the crystalline phase of the samples and confirmed the integration of Co_3_O_4_ and Co on the supports studied (zeolite, cerium oxide, and manganese oxide). Moreover, it was demonstrated that the addition of PVP as a stabilizing agent improves the catalytic capacity of Co_3_O_4_, as it helps to obtain more homogeneous and smaller nanoparticles. However, for the Co catalyst synthesized using PVP, this effect was not observed as a result of partial oxidation of the material due to the presence of the stabilizing agent. Comparing the effect of the supports studied, the performance of CeO_2_ as support was more promising, which was accounted for by the presence of oxygen vacancies in CeO_2_ that promote carbon dioxide adsorption and activation. In addition, the higher catalytic activity of the catalyst supported by CeO_2_ can be attributed to the generation of favorable interfacial sites between CeO_2_ and Co_3_O_4_ for the methanol synthesis reaction. 

## Figures and Tables

**Table 1:** Element quantification with EDX of Co_3_O_4_/MnO_2_ material.

Element	Weight (%)	Atomic (%)
C	28.60	43.21
O	38.52	43.68
Mn	14.38	4.75
Co	14.15	4.36
Others	3.81	3.58

**Table 2:** Element quantification with EDX of Co_3_O_4_/CeO_2_ material.

Element	Weight (%)	Atomic (%)
C	5.44	16.78
O	24.06	55.74
Co	21.43	13.48
Ce	48.46	12.82
Others	0.61	1.18

**Table 3:** Element quantification with EDX of Co_3_O_4_/zeolite material.

**Element**	**Weight (%)**	**Atomic (%)**
C	46.01	58.85
O	35.51	34.10
Si	2.12	1.16
Al	2.25	1.28
Co	11.83	3.08
Others	2.28	1.53

**Element**

**Weight (%)**

**Atomic (%)**

## Data Availability

Data are contained within the article.
